# Novel α-Amylase Inhibitor Hemi-Pyocyanin Produced by Microbial Conversion of Chitinous Discards

**DOI:** 10.3390/md20050283

**Published:** 2022-04-23

**Authors:** Thi Hanh Nguyen, San-Lang Wang, Anh Dzung Nguyen, Manh Dung Doan, Thi Ngoc Tran, Chien Thang Doan, Van Bon Nguyen

**Affiliations:** 1 Institute of Biotechnology and Environment, Tay Nguyen University, Buon Ma Thuot 630000, Vietnam; nguyenhanh2208.tn@gmail.com (T.H.N.); nadzung@ttn.edu.vn (A.D.N.); dmdung@ttn.edu.vn (M.D.D.); 2 Department of Science and Technology, Tay Nguyen University, Buon Ma Thuot 630000, Vietnam; ttngoc@ttn.edu.vn (T.N.T.); dcthang@ttn.edu.vn (C.T.D.); 3 Department of Chemistry, Tamkang University, New Taipei City 25137, Taiwan; 4 Life Science Development Center, Tamkang University, New Taipei City 25137, Taiwan

**Keywords:** α-amylase inhibitors, diabetes, hemi-pyocyanin, marine discards, microbial conversion

## Abstract

α-Amylase inhibitors (aAIs) have been applied for the efficient management of type 2 diabetes. The aim of this study was to search for potential aAIs produced by microbial fermentation. Among various bacterial strains, *Pseudomonas aeruginosa* TUN03 was found to be a potential aAI-producing strain, and shrimp heads powder (SHP) was screened as the most suitable C/N source for fermentation. *P. aeruginosa* TUN03 exhibited the highest aAIs productivity (3100 U/mL) in the medium containing 1.5% SHP with an initial pH of 7–7.5, and fermentation was performed at 27.5 °C for two days. Further, aAI compounds were investigated for scaled-up production in a 14 L-bioreactor system. The results revealed a high yield (4200 U/mL) in a much shorter fermentation time (12 h) compared to fermentation in flasks. Bioactivity-guided purification resulted in the isolation of one major target compound, identified as hemi-pyocyanin (HPC) via gas chromatography-mass spectrometry and nuclear magnetic resonance. Its purity was analyzed by high-performance liquid chromatography. HPC demonstrated potent α-amylase inhibitory activity comparable to that of acarbose, a commercial antidiabetic drug. Notably, HPC was determined as a new aAI. The docking study indicated that HPC inhibits α-amylase by binding to amino acid Arg421 at the biding site on enzyme α-amylase with good binding energy (−9.3 kcal/mol) and creating two linkages of H-acceptors.

## 1. Introduction

The fishery processing industry has been producing increasingly large amounts of byproducts, with the globally reported accessible waste of 27.85 million tons per year [1]. Among various fishery wastes, marine chitinous wastes (MCWs) including crab shells, squid pens, shrimp shells, and shrimp heads are abundantly available as fishery byproducts. Recently, these materials have been extensively used for the extraction and bioproduction of many active compounds [2]. MCWs have been utilized as carbon/nitrogen (C/N) sources for fermentation to produce various added valuable compounds such as enzymes, exopolysaccharide, chitosan oligomer, and *N*-acetyl-d-glucosamine [3,4], antioxidant, anti-NO, and anti-cancer compounds [5,6,7], biofertilizers [8], insecticidal materials [9], dye, and biosorbents [10]. MCWs have also been used for the extraction of astaxanthin, β-carotene esters, and essential amino acids [11,12]. Recently, MCWs were extensively used for the bioproduction of α-glucosidase inhibitors. In this work, MCWs were investigated for the production of α-amylase inhibitors (aAIs; anti-diabetic compounds) via microbial fermentation. 

Diabetes mellitus is a global health issue that significantly reduces the quality of life and health of people [13]. Among the two major types of diabetes mellitus, type 2 (T2D) accounts for 90% of diabetic cases [14]. In recent years, natural carbohydrate digestive enzyme inhibitors (CDEIs), including α-glucosidase inhibitors and aAIs, have been proved as a potentially effective therapy for the management of T2D [15,16,17,18]. Though some commercial inhibitors such as acarbose, voglibose, and miglitol are available, the use of these commercial drugs may cause some side effects; thus, the investigation of natural sources of inhibitors with improved safety is required [18]. 

CDEIs may be obtained from various natural sources including medicinal plants [16,18,19,20,21]. However, herbal CDEIs are difficult to obtain in large amounts [22,23,24]. Microbial fermentation is another approach to produce natural compounds including CDEIs [2,3,5,17,22]. In our previous works, we reported the production and purification of α-glucosidase inhibitors from microbes [17,24] and extensively used MCWs as C/N sources for the production of α-glucosidase inhibitors via fermentation [7,25,26,27,28,29,30,31]. Given our past research on recycling organic wastes into value-added anti-diabetic compounds, in this study, we investigated the use of CDEIs for the cost-effective production of aAIs via microbial fermentation. The production of aAIs was further scaled up in a 14 L-bioreactor system, and the major active compounds were isolated and identified. Then, a docking study was performed to elucidate the interaction of the inhibitor with the target enzyme. 

## 2. Results and Discussion

### 2.1. Screening Active α-Amylase Inhibitors Producing Strain

More than 100 bacterial strains obtained from our previous studies were assessed by fermentation to produce aAIs. Of these, the supernatants of seven strains showed positive anti-α-amylase activity (Table 1), with inhibition values ranging from 47–89%. Three bacterial strains, i.e., *Bacillus cereus* RB.DS.05, *Pseudomonas aeruginosa* TUN03, and *B. atrophaeus* H10, demonstrated the highest inhibition values (approximately 90%). For further confirmation of the results, the activities of the supernatants were tested at various dilutions and expressed as productivity (U/mL). *P. aeruginosa* TUN03 indicated the highest activity in the supernatant, with a productivity of 2430 U/mL. Thus, this strain was chosen for further investigation.

The use of *Pseudomonas aeruginosa* has been reported in agriculture [32,33,34,35], environment management [36,37], and for the production of secondary metabolites such as rhamnolipid, vanillin, enzymes, pigments, and plant promoting compounds with a wide range of applications [38,39,40,41,42,43]. Based on our recent review of published studies, there is no report on the use of this genus for the production of aAIs via fermentation. Thus, the finding of α-amylase inhibitory activity in this strain enriches the bioactivity catalog of *P. aeruginosa*.

### 2.2. Production of α-Amylase Inhibitors via P. aeruginosa TUN03 Fermentation

To establish the fermentation process to produce aAIs with optimal productivity, some factors, including the effect of C/N sources and culture conditions, were examined. Then, aAIs were scaled-up for production in a 14 L-bioreactor system. 

#### 2.2.1. The Effect of C/N Sources on aAI Production by *P. aeruginosa* TUN03

**Figure 1:**
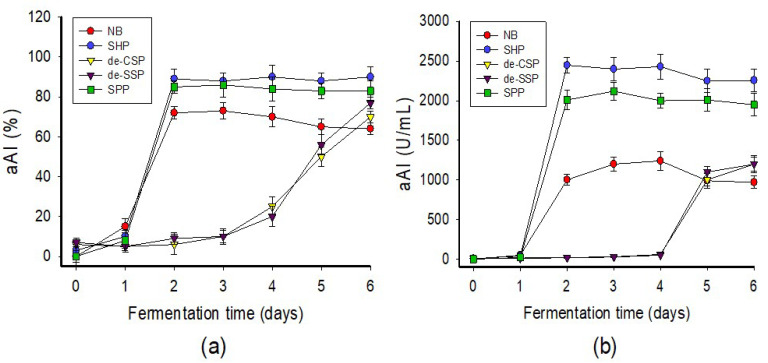
Screening of suitable C/N sources for fermentation. The C/N sources were used at 1% in fermentation at 30 °C, 150 rpm (shaking speed) for six days. The α-amylase inhibitory activity of the fermented culture broth was tested daily and expressed as % (**a**) or U/mL (**b**).

Various marine chitinous materials such as squid pen powder (SPP), shrimp head powder (SHP), demineralized shrimp shell powder (de-SSP), demineralized crab shell powder (de-CSP), and a commercial medium nutrient broth (NB) were used as the sole C/N source of *P. aeruginosa* TUN03 for fermentation and comparison of aAI production. As shown in Figure 1a, the medium containing SHP, SPP, and NB showed the highest activity on day 2 of fermentation, with respective inhibition values of 88, 85, and 72%. For better clarity of the results, the activity was also calculated and expressed as productivity (U/mL). The result presented in Figure 1b indicates that SHP was the most suitable C/N source and provided the highest productivity (2448 U/mL). Therefore, this substrate was chosen for the cost-effective production of aAIs in further experiments. Bacterial growth was also assessed but showed no correlation with aAI production (data not shown); thus, this factor was also not taken into account in the subsequent experiments. Shrimp industrial waste has been used for the production of chitin, protein, carotenoids, dye adsorbents, enzymes, and acetylcholinesterase inhibitor compounds [2,44,45,46]. Shrimp head is also used as fish meal [47,48]. Recently, shrimp head was extensively investigated as a C/N source for the production of α-glucosidase inhibitors [29,30,31]. In contrast to previous studies, in this work, we highlight the novel and potential use of this low-cost material as the sole C/N source for the production of aAIs via microbial fermentation. 

#### 2.2.2. The Effect of Culture Conditions on aAI Production by *P. aeruginosa* TUN03 

**Figure 2:**
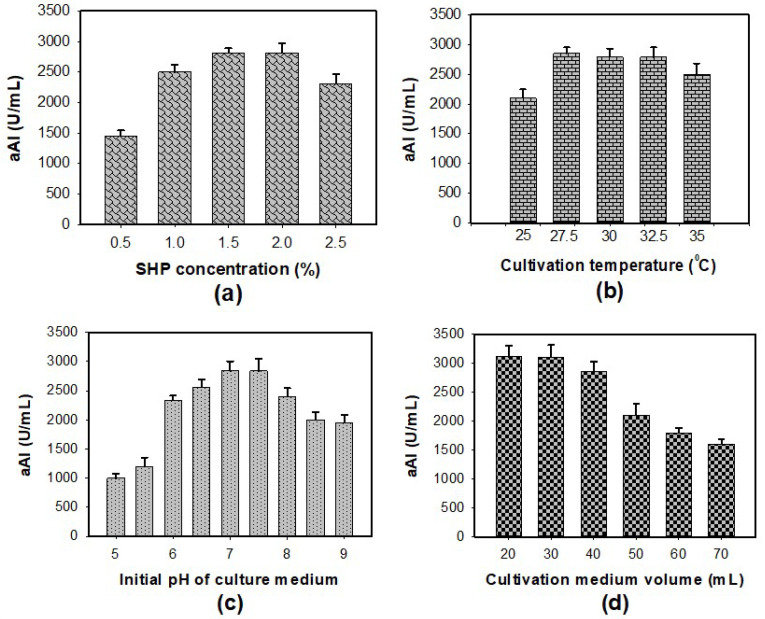
The effect of SHP concentration (**a**), cultivation temperature (**b**), initial pH of the culture medium (**c**), and cultivation medium volume (**d**) on aAI productivity in *P. aeruginosa* TUN03 fermentation.

To enhance aAI productivity, some culture conditions such as concentration of SHP, culture temperature, initial pH of the culture medium, and cultivation medium volume were investigated for their effect on aAI production by *P. aeruginosa* TUN03 fermentation (Figure 2). As presented in Figure 2a, suitable concentrations of SHP were determined to be 1.5–2%. In the interest of saving input material, 1.5% SHP was chosen for the next experiments. The data in Figure 2b indicate that *P. aeruginosa* TUN03 provided the highest aAI yield (2785–2850 U/mL) with a cultivation temperature in the range of 27.5–32.5 °C. As such, 27.5 ^°^C was set as the optimum cultivation temperature in order to minimize energy consumption. *P. aeruginosa* TUN03 was found to produce considerable amounts of aAIs with the initial pH of the culture medium of 7–7.5 (Figure 2c). The initial pH of the culture medium was applied for further investigations of cultivation medium volume. The result in Figure 2d shows the aAI production by *P. aeruginosa* TUN03 on a small scale (20–30 mL of medium in a 100 mL flask). Overall, *P. aeruginosa* TUN03 achieved the highest productivity of aAIs when a 30 mL medium containing 1.5% SHP was used with an initial pH of 7–7.75 in a 100 mL–flask and fermented at 27.5 °C for two days. 

#### 2.2.3. Scaled-Up of Production of α-Amylase Inhibitors Using a 14 L-Bioreactor System

**Figure 3:**
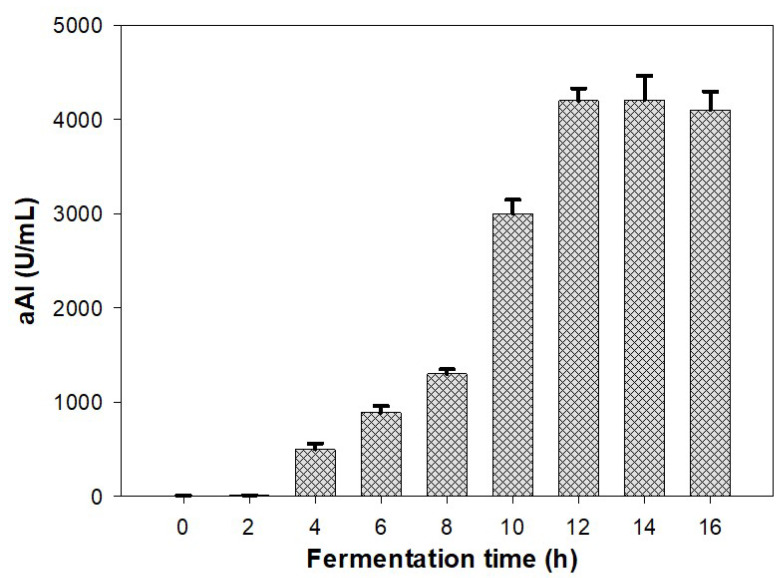
Scaled-up production of aAIs in a 14 L-bioreactor system. Standard errors (SE) are shown as error bars.

In microbial technology, reactor systems are considered strong tools for scaling-up the bioproduction of active secondary metabolites and significantly reducing cultivation time [2,49,50]. In this work, we utilized a 14 L-bioreactor system for fermentation to scale up the production of aAIs. As shown in Figure 3, aAIs were produced after 4 h of fermentation and reached the highest productivity, i.e., 4200 U/mL, at 12 h. These experimental data indicated that the scale-up in a bioreactor system yields significantly higher aAI productivity in a much shorter fermentation time than that in a flask. Previously, several studies reported the production of antidiabetic compounds via microbial fermentation [26,27,28,29,30,31]. However, in most of these works, compounds were produced on small scales in flasks. In this work, we attempted the large-scale production of antidiabetic compounds in a 14 L-bioreactor system and achieved positive outcomes.

### 2.3. Determination and Isolation of Major Active Compound from the Culture Broth

**Figure 4:**
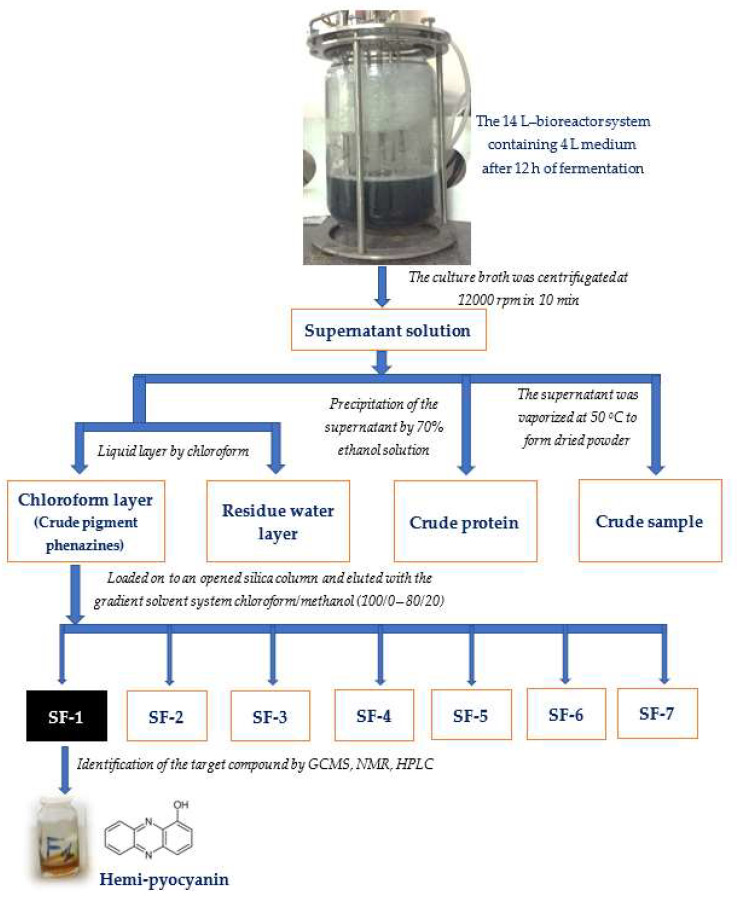
The process of preparation, purification, and identification of target compounds (α-amylase inhibitors).

Phenazine compounds are produced in significant quantities by *P. aeruginosa*. Thus, these compounds may play an important role in the bioactivities of *P. aeruginosa*. We found that the density of these pigments seemed to increase along with aAI production during *P. aeruginosa* TUN03 fermentation. On the other hand, earlier studies reported microbial aAIs as proteins [51,52]. Thus, to quickly predict the most active component, the supernatant was used to prepare some samples, including the crude pigment phenazines (the chloroform layer, named CPP), the residue water layer (named RWL), the crude protein (the supernatant was precipitated by ethanol, named CP) and the crude sample (the supernatant was vaporized to dried powder, named CS) to evaluate the inhibitory activity of α-amylase. The processes of preparation, purification, and identification of the target compounds are summarized in Figure 4. 

**Figure 5:**
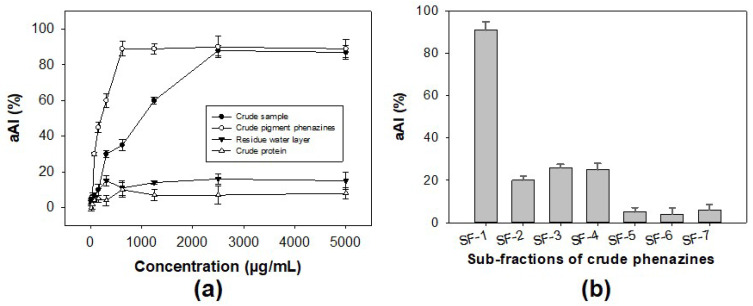
The bioactivity-guided purification process of α-amylase inhibitor compound. The α-amylase inhibitory activity of some major portions extracted from a crude sample tested at various concentrations (**a**) and sub-fractions of the crude pigment phenazine portion tested at a concentration of 150 µg/mL (**b**).

**Figure 6:**
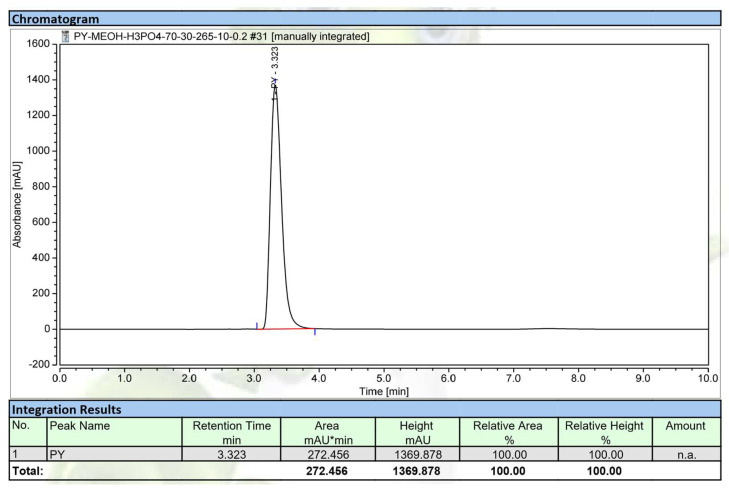
The HPLC profile of the purified compound, hemi-pyocyanin. The UV detection wavelength was 292 nm.

As shown in Figure 5a, the protein potion and the residue water layer showed no activity, while the pigment phenazines displayed high levels of activity, with a maximum inhibition of 89% (at a concentration of 1 mg/mL), which is much higher than that of a crude sample with maximum inhibition of 88% (at a concentration of 4 mg/mL). The results indicated that pigment phenazines were the major α-amylase inhibitor compounds in the sample. Thus, this portion with pigments was further separated using an opened silica column, and seven sub-fractions (SF-1, SF-2, SF-3, SF-4, SF-5, SF-6, and SF-7) were obtained. Then, the activity of these substrates was tested, and is presented in Figure 5b. The component SF-1 was found to exhibit the strongest activity (91%), while components SF-2, SF-3, and SF-4 displayed inhibition values under 26%, and SF-6 and SF-7 demonstrated very weak activity. The active phenazine compound SF-1 obtained in the yellow form was identified based on gas chromatography-mass spectrometry (GCMS) and nuclear magnetic resonance (NMR, including ^1^H-NMR, ^13^C-NMR, DEPT 135, DEPT 90, and DEPT 45) analyses; the spectra are presented in the Supplementary Materials, Figures S1–S6. This compound was further confirmed to possess the high level of purity achieved using high-performance liquid chromatography (HPLC) profiles (Figure 6).

As shown in Figure S1A, a major peak appeared with a retention time of 13.09 in the GC profile of the purified component SF-1. For further analysis, the mass of this peak was compared to those of known compounds in the chemical bank of the GCMS system (Figure S1B). On this basis, it was assumed that this compound was hydroxyphenazine, with the chemical formula C_12_H_8_N_2_O. The chemical structure of this compound was further identified based on an analysis of its NMR spectrum. Compound SF-1 was obtained as a yellow amorphous powder. ^1^H-NMR (600 MHz, CDCl_3_) δH: 8.25 (1H, m) δH: 8.21–8.25 (2H, m), δH: 7.76–7.89 (4H, m), δH: 7.24–7.26 (1H, m) (Figure S2). ^13^C-NMR (151 MHz, CDCl_3_) δC: 151.7, 144.3, 144.5, 141.2, 134.7, 131.9, 130.8, 130.5, 129.7, 129.2, 119.9 and 108.9 (see Figure S3). 

The ^1^H-NMR chemical shift of SF-1 indicated that 8 H atoms with δH from 7.0 to 8.5 ppm should be in the aromatic rings. Of these, the H with δH: 8.25 should be assigned to be the H atom of the hydroxyl group, while the other seven H atoms should be located in aromatic rings at 2, 3, 4, 6, 7, 8, 9 with δH: 8.21–8.25 (2H, m), δH: 7.76–7.89 (4H, m), δH: 7.24–7.26 (1H, m), respectively.

The ^13^C-NMR of SF-1 provided the signals of 12 C. Of these, the C atom (δC: 151.7) was assigned to the C of hydroxyl group (C-OH) in the aromatic rings, while other C atoms were assigned to aromatic rings at 2, 3, 4, 4a, 5a, 6, 7, 8, 9, 9a, 10a with δC: 144.3, 144.5, 141.2, 134.7, 131.9, 130.8, 130.5, 129.7, 129.2, 119.9 and 108.9, respectively. In addition, the ^13^C-NMR chemical shift coupling with spectra of DEPT 135, DEPT 90, and DEPT 45 (Figures S4–S6) also indicated that this compound contained seven groups of CH and 5 C. These observations allowed us to identify this compound as hemi-pyocyanin (also known as 1-hydroxyphenazine); the NMR spectra of this compound corresponded to that of 1-hydrophenazine, identified in an earlier report [53]. 

**Figure 7:**
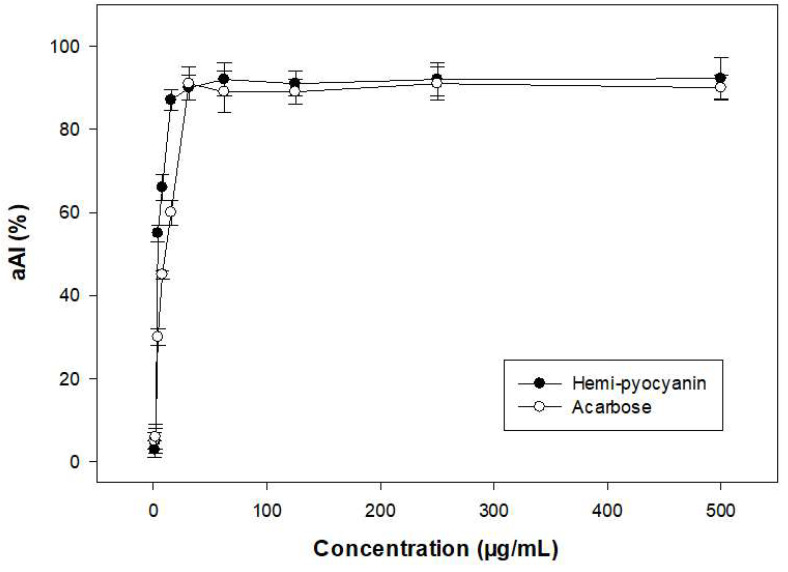
α-amylase inhibitory activity of purified hemi-pyocyanin and acarbose.

To check the purity grade of the isolated compound, HPLC was performed, and the purified HPC (SF-1) also appeared as a clear single peak (Figure 6). The purity of the obtained peak (HPC) was found to be 100% and 97.8% by HPLC using a UV detector and GCMS using an MS detector (ITQ 900), respectively. These results confirmed the high purity of the HPC. This compound was then further tested in detail for aAI activity (Figure 7). For comparison, this active compound and acarbose were tested at various concentrations. HPC showed potential α-amylase inhibition with maximum inhibition and IC_50_ values of 92.2% and 3.1 µg/mL, respectively (Figure 7). The activity of this compound was comparable to that of acarbose, a commercial antidiabetic drug with maximum inhibition and IC_50_ values of 91% and 4.2 µg/mL, respectively. It has been reported that HPC has potential medicinal effects, such as anti-cancer activity against cell lines 1321N1 and A549 [54,55], antibacterial activity against human pathogenic bacterial strains *Candida albicans*, *Aspergillus fumigatus*, *Escherichia coli*, *Xanthomonas campestris* pv. *vesicatoria* [55,56,57], and anti-inflammatory activities [58]. HPC has also reportedly been used in agriculture against various fungal and bacterial strains [53,59]. However, to the best of our knowledge and as per our review of the literature, no report has been published to date on the α-amylase inhibitory activity of HPC. Thus, the new finding in this work may facilitate the novel use of HPC in medicines. 

Post-prandial hyperglycemia has been recognized as playing important role in the development of T2D, i.e., by controlling the levels of plasma glucose required to delay or prevent T2D [60]. The compound has been suggested as a safe and complementary treatment for T2D. The inhibition of glycosidase with, for example, a-amylase and a-glucosidase, which convert dietary starch into glucose, has been considered a method for controlling plasma glucose [61,62]. It is reported that the a-(1,4)-glycosidic linkages of starch are degraded by α-amylase to produce maltose and glucose; then, maltose molecules are further hydrolyzed to glucose by α-glucosidase before entering the blood via intestinal epithelial absorption. Therapy using glycosidase inhibitors was considered a more effective method than controlling insulin secretion for convenience and economic reasons, as well as the reduced side-effects [63]. In addition, aAIs are known to prevent dietary starches from being absorbed by the human body by acting as starch blockers. As such, they have also been used as slimming pills [64]. In this study, HPC demonstrated novel potential inhibition against a mammalian α-amylase with a comparable effect to that of acarbose, a commercial antidiabetic drug. Thus, HPC could be a good candidate for the management of T2D and obesity. 

### 2.4. Docking Study Showing the Interaction of Hemi-Pyocyanin at the Binding Site of α-Amylase

**Figure 8:**
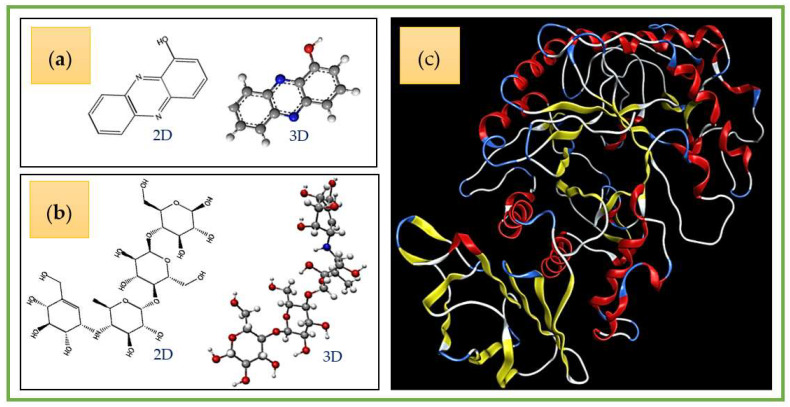
The 2-D and 3-D structures of hemi-pyocyanin (**a**) and acarbose (**b**), and the crystal structure of α-amylase (**c**).

A docking study was performed to explore the interactions of ligands (HPC and acarbose) and the target enzyme α-amylase. Before examining docking, the structures of ligands and a protein enzyme were prepared using ChemBioOffice 2018 and MOE-2015.10 software. The molecular structures are shown in Figure 8.

**Figure 9:**
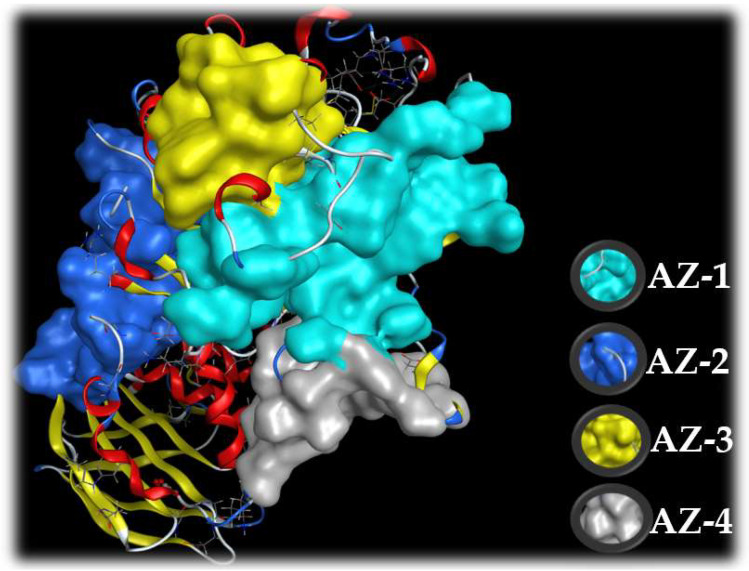
The four active zones (AZ) on the target enzyme, α-amylase, based on MOE output data using the site finder function.

The active zones of the ligands on the target protein were determined using the site finder function in MOE. The output data of MOE indicated that there are active zones (AZ-1, AZ-2, AZ-3, and AZ-4) of the ligands on the enzyme α-amylase (Figure 9). Of these, the HPC inhibitor was found to be the most active in zone AZ-2, while acarbose demonstrated potential inhibition against α-amylase in active zone AZ-1. AZ-1 contains up to 36 amino acids (Val49, Val50, Val51, Thr52, Asn53, Trp58, Trp59, Tyr62, Gln63, His101, Gly104, Ser105, Gly106, Ala107, Ala108, Tyr151, Leu162, Val163, Gly164, Leu165, Arg195, Asp197, Ala198, Lys200, His201, Glu233, Val234, Ile235, Glu240, His299, Asp300, Arg303, Gly304, His305, Gly306 and Asp356), while AZ-2 contains up to 28 (Ala3, Pro4, Gln5, Thr6, Gln7, Ser8, Gly9, Arg10, Thr11, Arg92, Trp221, Phe222, Pro223, Arg252, Ser289, Asp290, Pro332, Tyr333, Gly334, Phe335, Thr336, Arg398, Val401, Asp402, Gly403, Gln404, Pro405 and Arg421). Thus, these active zones were chosen to assess the docking performance of acarbose and hemi-pyocyanin, respectively. 

In the docking simulation, the Root Mean Square Deviation (RMSD) value was used to define whether the interactions between the ligand and the target protein enzyme were accepted or not. When RMSD reached a value higher than 3.0 Å, the interaction was considered as non-significant. Interaction is widely accepted when this value is under 2.0 Å [65]. As shown in Table 2, both HPC and acarbose are bound with α-amylase with RMSD values of 1.68 and 1.59, respectively, indicating that these two ligands successfully bound to the target enzyme. To evaluate the potential inhibition of the ligands toward the target enzyme, a docking score (DS) was used. A ligand is defined as a potent inhibitor when it has a binding energy value of under −3.20 kcal/mol [66]. In this work, HPC and acarbose were found to bind with α-amylase to generate DS values of −9.3 and −12.1 kcal/mol, respectively, which are much lower than −3.20 kcal/mol. Thus, this result confirmed that both these compounds are potential α-amylase inhibitors.

**Figure 10:**
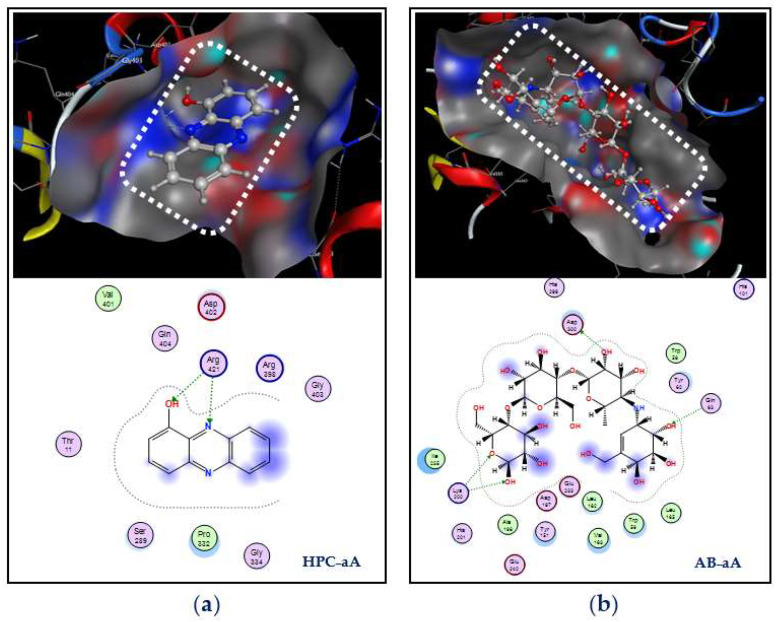
Docking study of hemi-pyocyanin (HPC) and acarbose with the α-amylase enzyme. The interactions and binding of the ligands HPC and acarbose with the α-amylase enzyme are labeled as HPC–aA (**a**) and AB–aA (**b**), respectively.

Simultaneous interactions of ligands with the target enzyme were examined; the details are presented in Figure 10. HPC interacts with α-amylase at active zone AZ-2 via the creation of two linked H-acceptors with amino acid Arg421. These two linkages are formed by the connection of the receptor group –NH2 of Arg421 to O1 and N2 of HPC with a distance and energy binding of 3.20 Å, −0.7 kcal/mol, and 3.1 Å, −1.7 kcal/mol, respectively. Acarbose was found to bind at active zone AZ-1 on α-amylase via the formation of four linkages, including one H-donor and three H-acceptor linkages. Asp300 and Gln63 in the active zone were found to connect with O6 and O10 of acarbose to form one linkage H-donor and one linkage H-acceptor with distances and energy binding of 2.65 Å, −2.8 kcal/mol, and 3.05 Å, −1.1 kcal/mol, respectively, while the amino acid Lys200 connected with O8 and O16 of acarbose to generate two H-acceptor linkages with a distance and energy binding of 3.37 Å, −1.9 kcal/mol, and 3.04 Å, −2.1 kcal/mol, respectively.

Based on the in vitro activity and a docking study on the inhibition against α-amylase, the biosynthesized and purified HPC was shown to be a potential α-amylase inhibitor with activity comparable to that of acarbose. As such it may be recommended as a potent candidate antidiabetic drug. However, further studies using various animal models and clinical studies are required for the development of this potential compound into an antidiabetic drug.

## 3. Materials and Methods

### 3.1. Materials

Bacterial strains were obtained from our previous works [43,67]. Discarded marine chitinous materials such as shrimp shells, shrimp heads, crab shells, and squid pens were obtained from Shin-Ma Frozen Food Co. (I-Lan, Taiwan). Porcine pancreatic α-amylase (type VI-B) and acarbose were purchased from Sigma Aldrich (St. Louis City, MO, USA). Shrimp shells and crab shells were demineralized as per the method presented in our previous study [68]. Silica gel (Geduran^®^ Si 60, size: 0.040–0.063 mm) was purchased from Merck Sigma Chemical Co (St. Louis City, MO, USA). The nutrient broth was purchased from Creative Life Science Co., Taipei, Taiwan, and some solvents used in this work were obtained from Sigma Aldrich (St. Louis City, MO, USA).

### 3.2. Methods

#### 3.2.1. α-Amylase Inhibitor Production via Microbial Fermentation Experiments

Screening of Active α-Amylase Inhibitors Produced from Discarded Marine Chitinous Material Which Was Suitable for Fermentation

The bacterial strains were obtained as in our previous works, and were examined for use in the fermentation of SHP. A liquid medium of 30 mL (initial pH 6.8) containing 1% SHP, 0.1% K_2_HPO_4_, and 0.05% MgSO_4_·7H_2_O in a 100 mL-Erlenmeyer flask was fermented using different strains at 30 °C with a shaking speed of 150 rpm for four days. Then, the supernatant was harvested by centrifugation at 12000 rpm for 10 min and used to detect the inhibitory activity of α-amylase. The most active strain, *P. aeruginosa* TUN03, was used for further experiments to screen the most suitable C/N sources. A total of four marine chitinous discards, i.e., SPP, SHP, de-SSP, and de-CSP, and a commercial medium nutrient broth (NB) were used as the sole C/N sources. These C/N sources were used at 1% concentrations in a basal salt medium of 0.1% K_2_HPO_4_ and 0.05% MgSO_4_·7H_2_O. Fermentation was performed at 30 °C with a shaking speed of 150 rpm for six days. The supernatant was harvested on a daily basis and used for the detection of activity. SHP was found to be the most suitable material for fermentation and was used for our subsequent investigation. 

The Effect of Culture Conditions on aAI Production by *P. aeruginosa* TUN03

To achieve higher aAI productivity, some culture conditions were tested. A 30 mL liquid medium in a 100 mL-Erlenmeyer flask containing different concentrations of SHP (0.5, 1, 1.5, 2, and 2.5%), 0.1% K_2_HPO_4_, and 0.05% MgSO_4_·7H_2_O was fermented by *P.s aeruginosa* TUN03 at 30 °C with a shaking speed of 150 rpm. The activity of the supernatant was tested after two days of fermentation. SHP (1.5%) was chosen for further experimentation to investigate the effect of culture temperature (25, 27.5, 30, 32.5, and 35 °C), the initial pH of the culture medium (5, 5.5, 6, 6.5, 7, 7.5, 8, 8.5, and 9), and cultivation medium volume (20, 30, 40, 50, 60, and 70 mL). The following experiments were designed based on the optimal conditions achieved in previous experiments.

Scale-Up of Production of α-Amylase Inhibitors Using a 14 L-bioreactor System

The optimal culture conditions investigated in the aforementioned experiments were applied to scale up the production of aAIs in a 14 L-bioreactor system (the 14 L BioFlo/CelliGen 115 bioreactor system—Eppendorf North America, Enfield, Connecticut, CT, USA). First, 500 mL bacterial seeds were pre-incubated in 250 mL flasks at 30 °C for two days and then transferred to the fermenter with 4.5 L of medium containing 1.5% SHP, 0.1% K_2_HPO_4_ and 0.05% MgSO_4_·7H_2_O, with an initial pH of 7. Fermentation was performed at 2.75 °C at a shaking speed of 150 rpm, with a dissolved oxygen content of 1.0 vvm for 16 h. Sampling was done every 2 h. The supernatant was harvested by centrifugation of the sample at 12,000 rpm for 10 min, and was used to detect aAI activity. 

#### 3.2.2. α-Amylase Inhibitory Activity Assay 

The inhibitory activity of α-amylase was assessed using the method of Bernfeld [69] with modifications. One hundred and fifty microliters of the α-amylase solution (0.25 U/mL) was mixed with 50 µL sample (supernatants or compounds at different concentrations) and kept at 37 °C for 10 min. Then, 200 µL soluble starch (0.25%) was added to start the reaction, which was kept at 37 °C for a further 20 min. The amount of reducing sugar produced by the action of the enzyme was measured at OD_540nm_. The enzymatic inhibitory activity was estimated using the following equation:

α-amylase inhibitory activity (%) = (C − E)/C × 100

where E is the optical density of the reaction containing the sample (inhibitor) and enzyme, and C is the optical density of the reaction containing enzyme and the same volume of distilled water instead of the sample [70]. The IC_50_ (µg/mL) and productivity (U/mL) were defined and calculated using the method presented by Nguyen et al. (2016) [71]. In this assay, the enzyme (0.25 U/mL) and substrate (0.25 % *w*/*v*) were both prepared in 10 mM CaCl_2_ solution in 20 mM Tris–HCl buffer at pH 7 before use.

#### 3.2.3. Extraction, Identification and Purity Confirmation of α-Amylase Inhibitors 

Extraction of α-amylase inhibitors: The culture broth obtained from the fermentation in the 14 L-bioreactor system was centrifuged at 12,000 rpm for 10 min to harvest the supernatant, which was further used to prepare some of the samples. The supernatant was separated from the chloroform layer to obtain the crude pigment phenazines (in the chloroform layer) and the residue water layer. The crude protein contained in the supernatant was obtained by precipitation with 70% ethanol solution. The supernatant was also vaporized at 50 °C to dried powder (crude sample). The crude pigment phenazines exhibited the most activity and were therefore further used to isolate the target components using an opened silica column (Geduran^®^ Si 60, size: 0.040–0.063 mm, 30 × 2 cm) with a gradient solvent system, i.e., chloroform/methanol (100/0—80/20), to obtain seven sub-fractions: SF-1, SF-2, and SF-3, (eluted in chloroform/methanol of 100/0), SF-4 (eluted in chloroform/methanol of 100/5), SF-5 and SF-6 (eluted in chloroform/methanol of 100/10), and SF-7 (eluted in chloroform/methanol of 100/20). Component SF-1 was found to be active, and was identified using GCMS and NMR. Its purity was further confirmed by HPLC analysis. 

Identification of α-amylase inhibitors: GCMS was conducted to test the presence of active phenazine compounds. Helium gas (99.999%) was used as a carrier gas at a constant flow rate of 1 mL/min, and 1 μL of an injection volume was employed (a split ratio of 10:1). The injector temperature was maintained at 250 °C, the ion-source temperature was 250 °C and the oven temperature was programmed to 70 °C (isothermal for 1 min), with an increase of 15 °C/min to 200 °C, ending with a 2 min isothermal at 200 °C. MS data were acquired at 70 eV, a scanning interval of 0.5 s, and fragments from 50 to 650 Da.

Purity confirmation of α-amylase inhibitors using HPLC: The compound was dissolved in methanol, and then filtered through a 0.22 µm membrane. The sample solution (2 µL) was injected into the HPLC system (Thermo-Ultimate 3000 UPLC system–ThermoScientific, Waltham, MA, USA) and separated by a column (Hypersil GOLD aQ C18 column, 150 mm × 2.1 mm, particle size: 3 µm) using the solvent systems of methanol/acidified 0.1% H_3_PO_4_ (70/30 *v*/*v*) with the flow rate of 0.2 mL/min. The UV detection wavelength was 265 nm. 

#### 3.2.4. Docking Study 

A docking study was performed following the steps described in our earlier reports [2,25].

Preparation of the target protein: The structural data of the protein enzyme (α-amylase) were obtained from the Worldwide Protein Data Bank. The 3-D structure was produced using MOE QuickPrep based on the positions of the ligand within 4.5 Å and the presence of important amino acids. The active zones were found using the site finder in MOE, and all of the water molecules were removed before the creation of the enzymic action zones. 

Preparation of ligands: The structures (2D and 3D) of HPC and acarbose were prepared using the ChemBioOffice 2018 software, and optimized using the MOE-2015.10 software with parameters of Force field MMFF94x, R-Field 1:80, cutoff, Rigid water molecules, space group p1, cell size 10, 10, 10, cell shape 90, 90, 90, and gradient 0.01 RMS kcal.mol^−1^A^−2^. 

Docking performance: Docking was performed on the HPC and acarbose ligands toward the target enzyme α-amylase using the MOE-2015.10 software. The output data, including energy biding (docking score, DS), RMSD, linkages types, amino acid compositions, distances, and the energy biding of each linkage, were recorded.

## 4. Conclusions

α-Amylase inhibitors (aAIs) have been used as an effective therapy for type-2 diabetes, which remains a global health issue. Though some commercial inhibitors are available, these drugs may cause side effects. Thus, the investigation of natural sources of inhibitors which are safe to use is required. The aim of this study was to evaluate the potential aAIs produced by microbial fermentation. *P. aeruginosa* TUN03 was selected as a potential aAI-producing strain, and SHP was found to be the most suitable C/N source for the production of aAIs via fermentation. In this way, high yields of aAIs were achieved. Further scaled up production of aAIs with higher yields and much shorter fermentation times via the utilization of a 14 L-bioreactor system was also attempted. The bioactivity-guided purification resulted in the isolation of one major active compound. This purified compound was identified as hemi-pyocyanin (HPC). Subsequently, a docking study was conducted to elucidate the interaction of this inhibitor with the target enzyme, α-amylase. This study provides some new findings, as summarized below:

- This study was the first to report on the use of discarded marine chitinous material for the cost-effective production of α-amylase inhibitor compounds via microbial fermentation.
- *P. aeruginosa* TUN03 was found to be a novel α-amylase inhibitor-producing strain.
- The production of α-amylase inhibitor compounds was successfully scaled up, i.e., in a 14 L-bioreactor system, achieving high productivity (4200 U/mL) in a short fermentation time (12 h).
- Hemi-pyocyanin, a major active compound purified from the culture broth, was identified as a new and potent α-amylase inhibitor.
- In a docking study, hemi-pyocyanin was found to bind to the target enzyme with a good docking score (−9.3 kcal/mol) via linking with amino acid Arg421 and the creation of two H-acceptor linkages.

The results of this work indicate that discarded shrimp heads are a valuable source material for the cost-effective bioproduction of a novel α-amylase inhibitor HPC which may be a good candidate for the development of new antidiabetic drugs. However, further studies using various animal models and clinical studies are required for the development of this compound into an antidiabetic drug. 

## Figures and Tables

**Table 1:** The α-amylase inhibitory activity of tested bacterial strains.

Bacterial Strains	α-Amylase Inhibitory Activity	
	Inhibition (%)	Productivity (U/mL)
*Bacillus megaterium* CC05	71 ± 2.1	-
*Acinetobacter baumannii* CC11	47 ± 0.9	-
*Bacillus marisflavi* BMT2	63 ± 1.3	-
*Bacillus cereus* RB.DS.05	89 ± 3.2	1750 ± 87.1
*Pseudomonas aeruginosa* TUN03	88 ± 2.7	2430 ± 106
*Bacillus acidicola* B14	56 ± 2.2	-
*Bacillus atrophaeus* H10	89 ± 4.3	1856 ± 112

Shrimp head powder was used as a C/N source for fermentation at 30 °C for four days. The supernatant was harvested by centrifugation at 12,000 rpm for 10 min and used to detect activity. (-): not determined.

**Table 2:** Docking simulation results of ligands binding with target enzyme α-amylase (aA).

Ligands (Inhibitors)	Symbol (Ligand-Protein)	RMSD (Å)	DS (kcal/mol)	Number of Linkages	Amino Acids Interacting with the Ligand [Distance (Å)/E (kcal/mol)/Linkage Type]
Hemi-pyocyanin (HPC)	HPC-aA	1.68	−9.3	2 linkages (H-acceptor)	Arg421 (3.20/−0.7/H-acceptor) Arg421 (3.10/−1.7/H-acceptor)
Acarbose (AB)	AB-aA	1.59	−12.1	4 linkages (1 H-donor and 3 H-acceptor)	Asp300 (2.65/−2.8/H-donor) Lys200 (3.37/−1.9/H-acceptor) Gln63 (3.05/−1.1/H-acceptor) Lys200 (3.04/−2.1/H-acceptor)

## Data Availability

Not applicable.

## Supplementary Materials

The following supporting information can be downloaded at: https://www.mdpi.com/1660-3397/20/5/283/s1, Figure S1. The gas chromatography-mass spectrometry (GCMS) profile of purified compound SF-1. The GC profile (A) and MS profile (B); Figure S2. 1H NMR spectrum of compound SF-1, measured in CDCl3 at 600 MHz; Figure S3. 13C NMR spectrum of compound SF-1, measured in CDCl3 at 151 MHz; Figure S4. DEPT 135 spectrum of compound SF-1, measured in CDCl3 at 151 MHz; Figure S5. DEPT 90 spectrum of compound SF-1, measured in CDCl3 at 151 MHz; Figure S6. DEPT 45 spectrum of compound SF-1, measured in CDCl3 at 151 MHz.
