# Supplementary material for: Novel α-Amylase Inhibitor Hemi-Pyocyanin Produced by Microbial Conversion of Chitinous Discards
Source: Mar Drugs. 2022 Apr 23;20(5):283. doi: 10.3390/md20050283 (PMC9171587; doi:10.3390/md20050283)
Supplement: Supplementary file 1 [file marinedrugs-20-00283-s001.zip › marinedrugs-1630266-supplementary.pdf]

Supplementary materials:

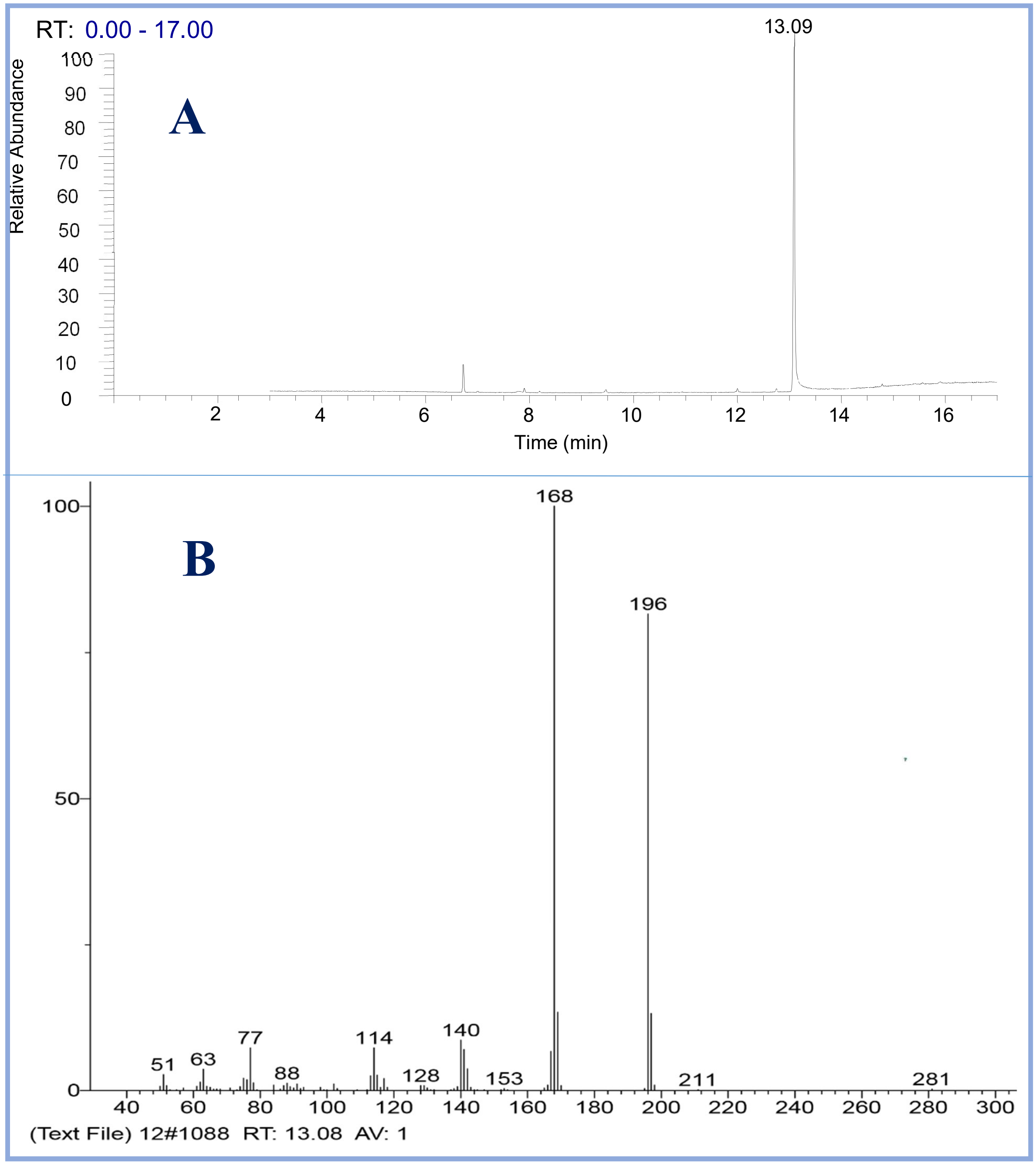

Figure S1. The gas chromatography-mass spectrometry (GCMS) profile of purified compound SF-1. The GC profile (**A**) and MS profile (**B**).

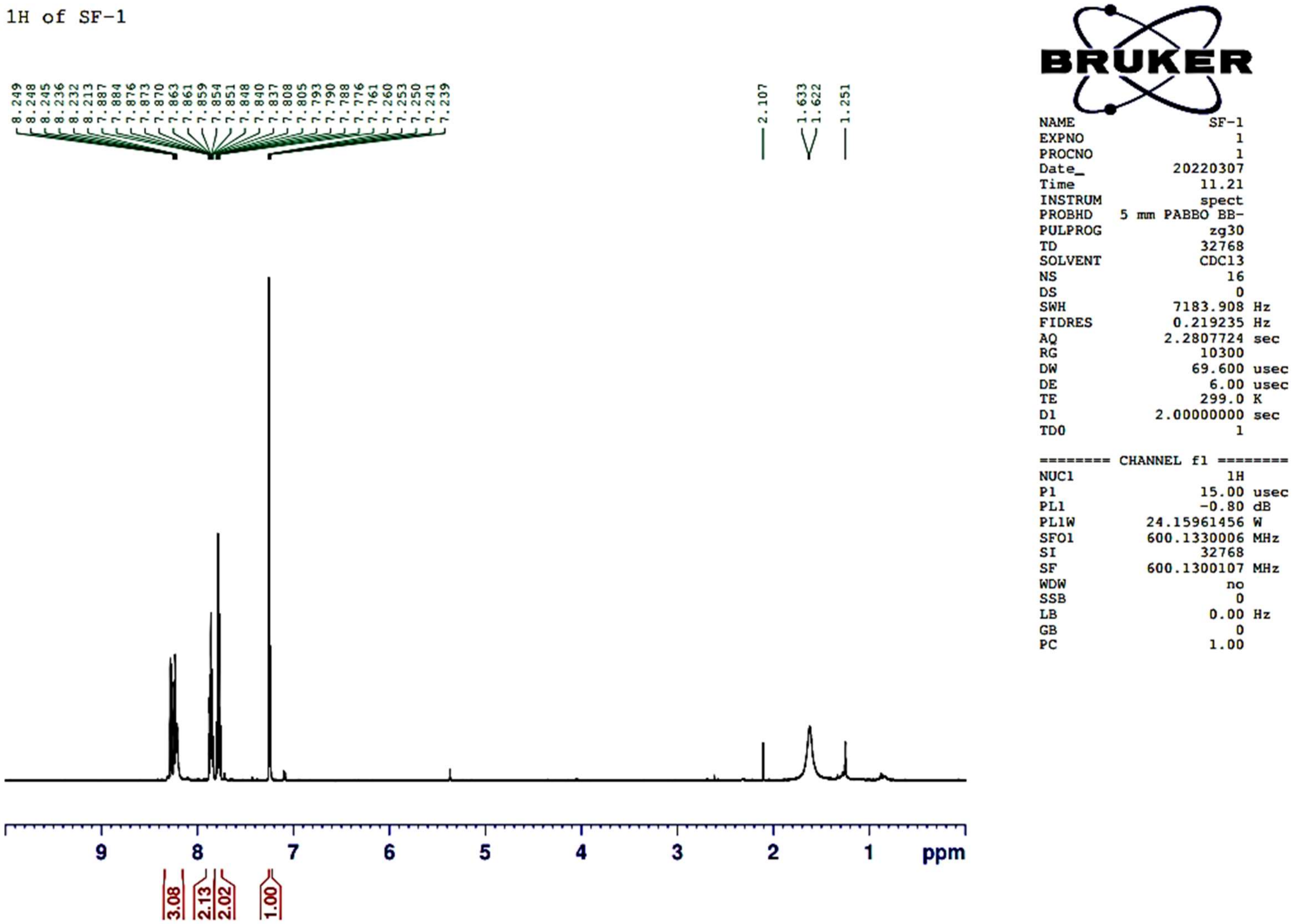

Figure S2. ^{1}H NMR spectrum of compound SF-1, measured in $CDCl_3$ at 600 MHz.

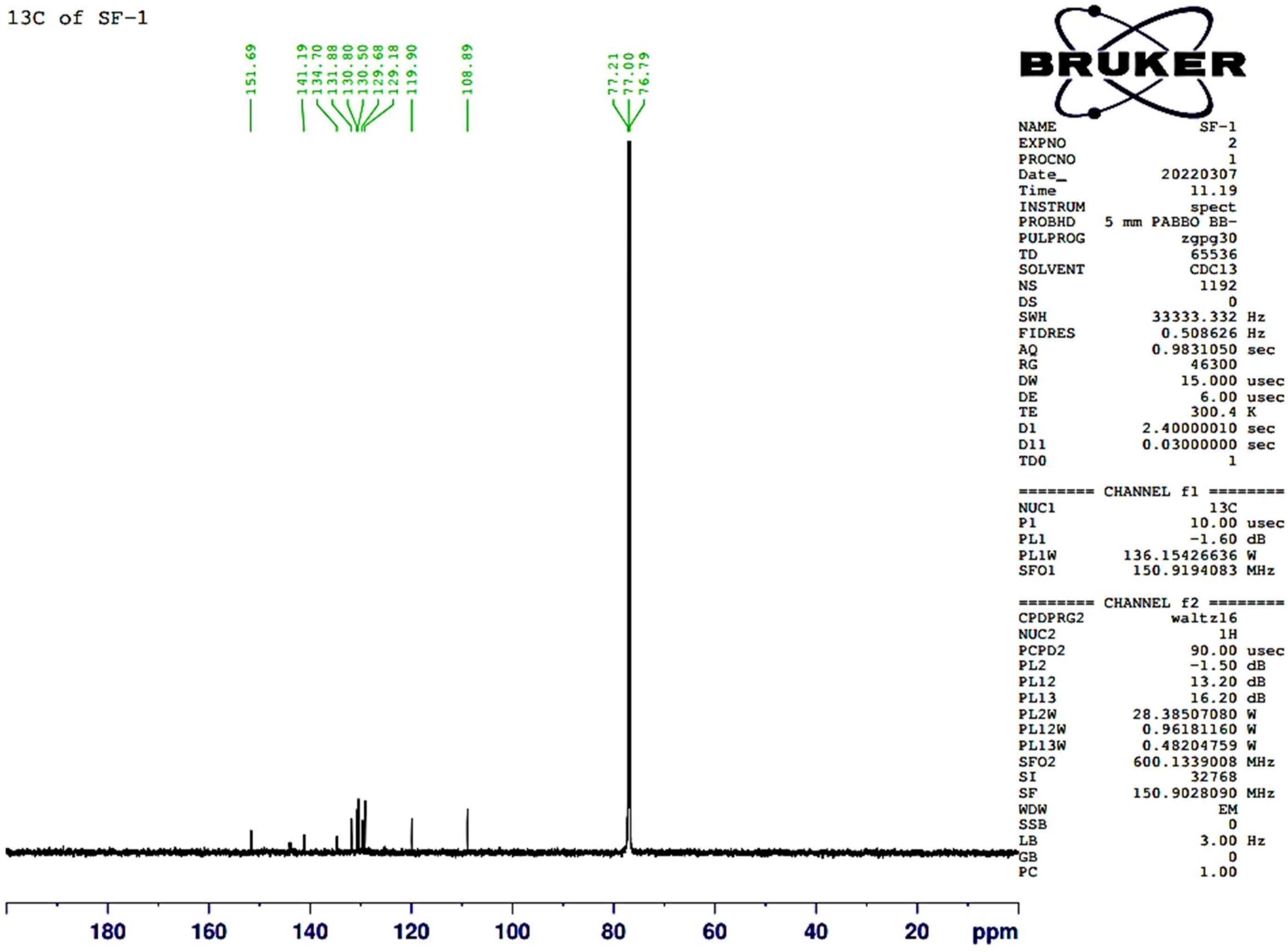

Figure S3. ^{13}C NMR spectrum of compound SF-1, measured in $CDCl_3$ at 151 MHz.

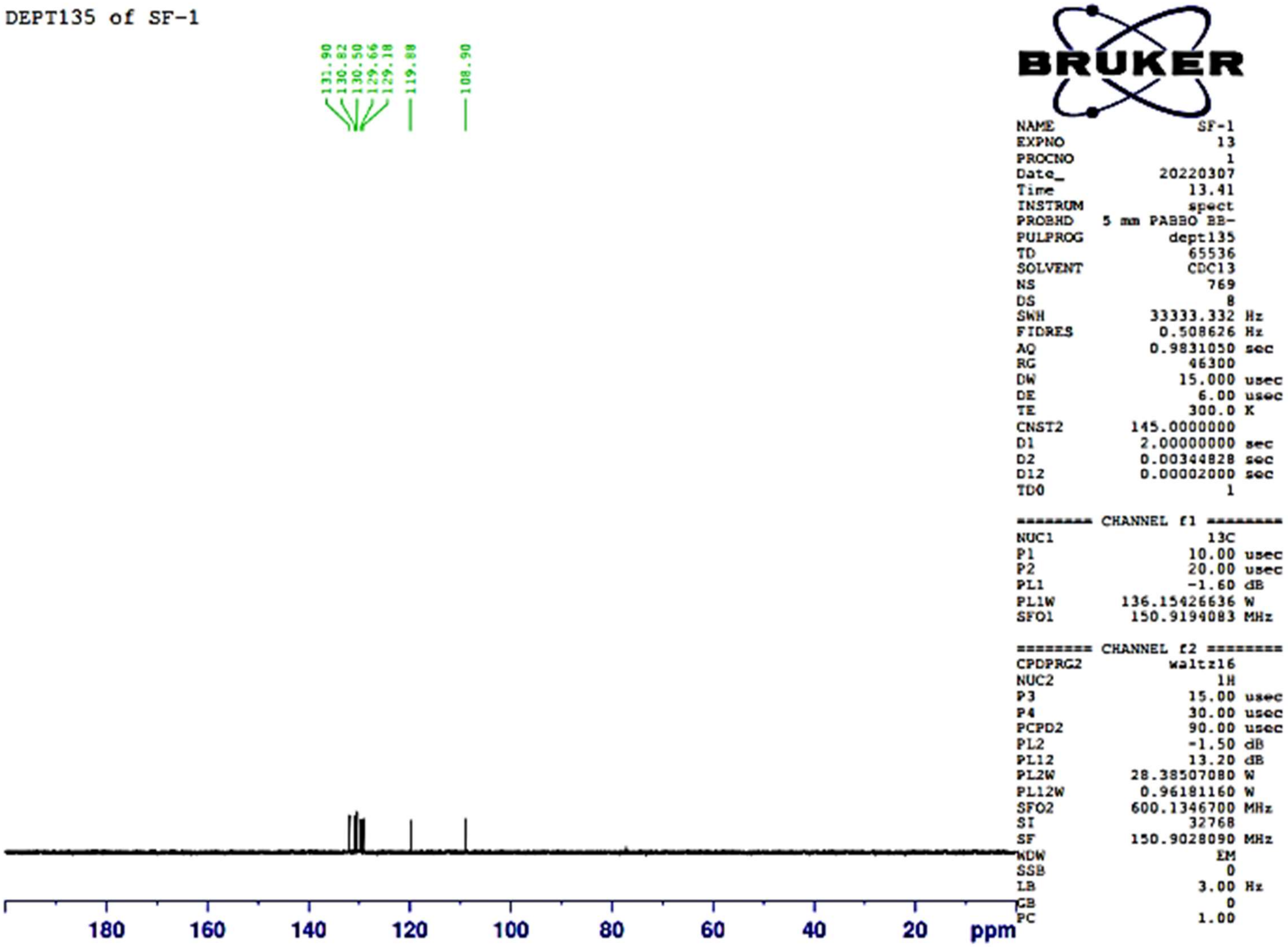

Figure S4. DEPT 135 spectrum of compound SF-1, measured in $CDCl_3$ at 151 MHz.

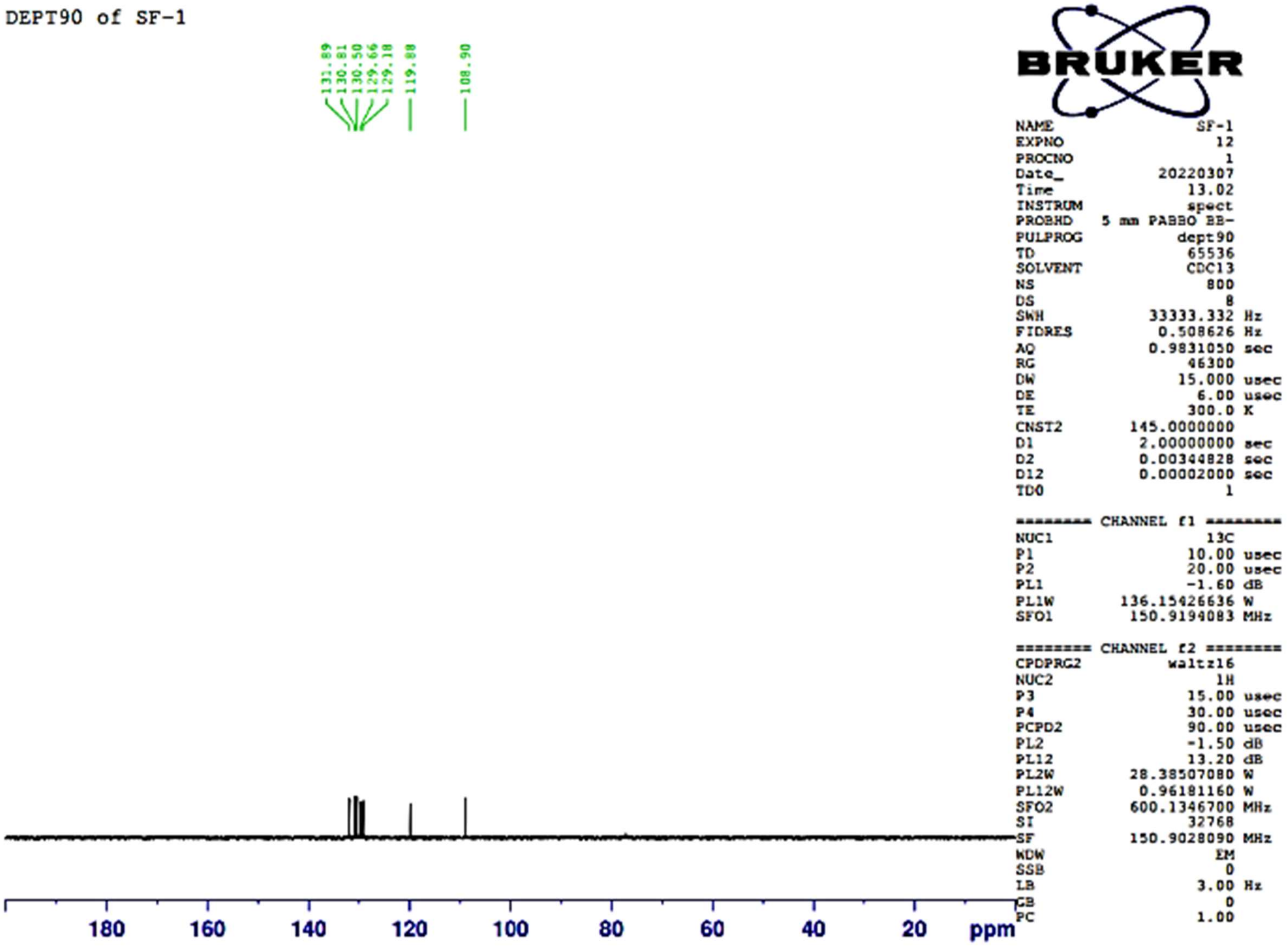

Figure S5. DEPT 90 spectrum of compound SF-1, measured in $CDCl_3$ at 151 MHz.

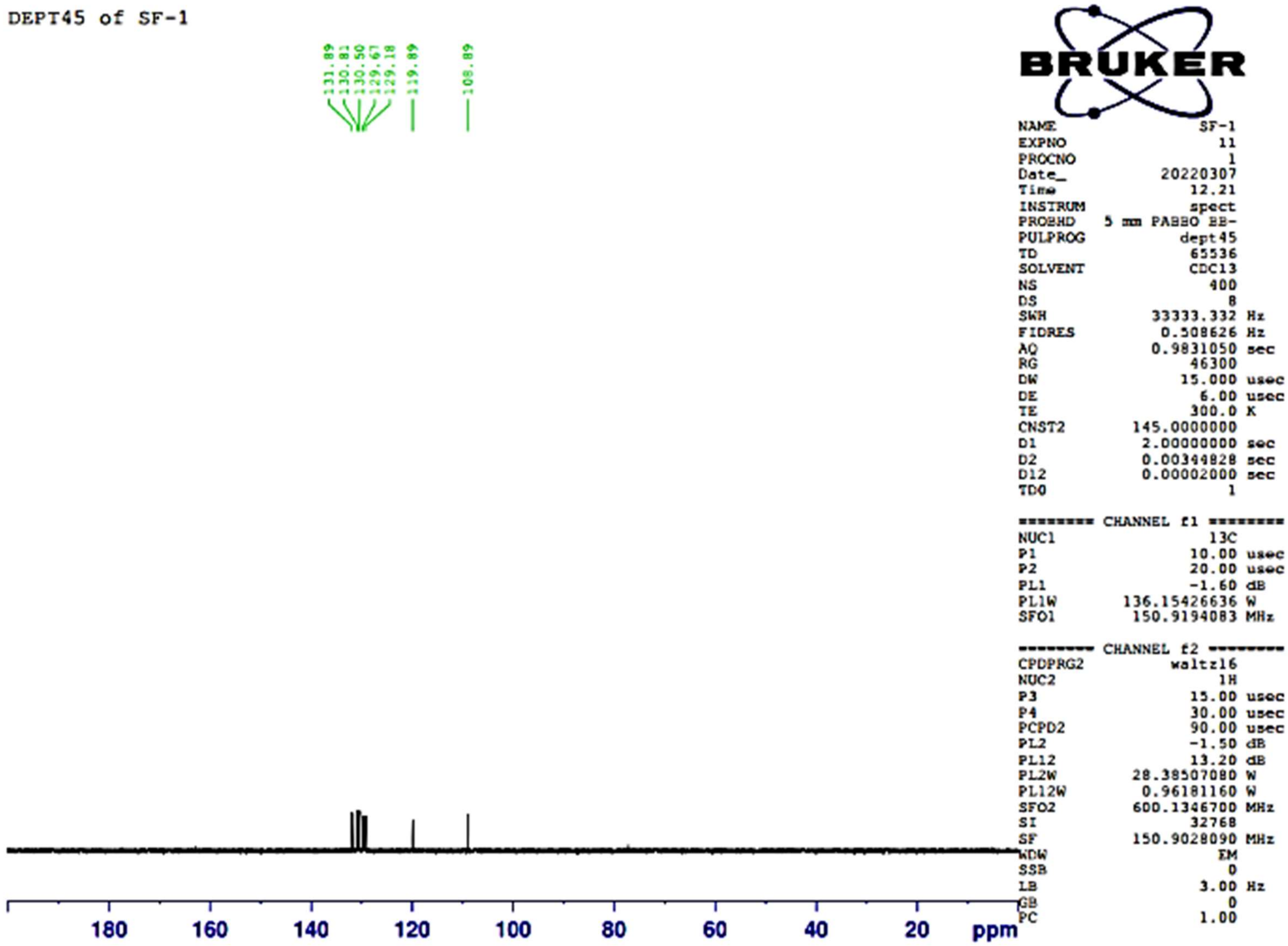

Figure S6. DEPT 45 spectrum of compound SF-1, measured in $CDCl_3$ at 151 MHz.
